# Determination of mandibular morphology in a TURKISH population with Down syndrome using panoramic radiography

**DOI:** 10.1186/s12903-019-0722-8

**Published:** 2019-02-26

**Authors:** Samed Satir

**Affiliations:** Akdeniz University, Faculty of Dentistry, Oral and Maxillofacial Radiology, Akdeniz University Dumlupinar Boulevard 07058 Campus Antalya, Antalya, Turkey

**Keywords:** Mandibular canal, Mandibular foramen, Down syndrome

## Abstract

**Background:**

Down syndrome (DS) is by far the most common known chromosomal disorder. Some characteristic features of DS are generalised growth deficiency, craniofacial abnormalities such as mandibular prognathism and underdevelopment of the midfacial region, dental abnormalities such as taurodontism and hypodontia. Individuals with DS have an increased prevalence of periodontal disease compared with age-matched control patients. The aim of the present study is to determine the morphologic features of the mandible among individuals with DS.

**Methods:**

Thirty-four DS patients and thirty four age- and gender-matched control subjects underwent panoramic radiography, which included measurement of the mandibular canal (MC), the mandibular foramen (MF), the mandibular ramus (MR), the distance from the MC to the mandibular lower border (C-MLB), and the distance between the MC and the alveolar crest upper limit (C-AUL). Patients were separated into two groups based on age: < 15 (*n* = 15) and ≥ 15 (*n* = 19). In order to determine whether the MF, MR, MC, C-AUL, and C-MLB scores differed according to the groups (DS and control), one-way multivariate analysis of covariance (MANCOVA) was applied in which gender and age were taken as covariates.

**Results:**

When the main effect according to the group was examined separately according to each measurement, the MF in the DS group was high with a moderate effect (*F* = 9207; *p* = 0.003). MR (*F* = 40,518; *p* < 0.001), MC (*F* = 23,747; *p* < 0.001), and C-AUL (*F* = 58,571; *p* < 0.001) in the DS group were lower with a larger effect. C-MLB did not significantly differ between the groups, and the effect size was quite low (*p* > 0.05).

**Conclusions:**

Mandibular canal morphology may exhibit anatomical variations in DS. The alveolar bone level may differ from non-DS due to growth development retardation and/or periodontal diseases.

## Background

Down syndrome (DS) is a chromosomal disorder with an estimated prevalence of 8–9 per 10,000 people in the USA [1]. According to a study in Denmark, the average life span for 64% of DS individuals is 50 years [2].

The height and head circumference of Turkish children with DS were lower compared to the normal population, and growth velocity was reduced, especially in early childhood and puberty [3]. Patients with DS typically present some craniofacial abnormalities such as a class III skeletal pattern and long lower anterior facial heights [4].

Individuals with DS have an increased prevalence of periodontal disease compared with age-matched control patients [5, 6]. It is stated that periodontal disorders begin early in DS children. Also, some studies in DS groups, including prepubertal children with DS, showed that alveolar bone loss was significantly higher in DS individuals than in the control group [7].

Although implant applications in patients with DS are usually performed under general anesthesia [8], local anesthesia procedures have also been reported in recent years [9, 10]. The vertical bone level on the mandible and the mandibular canal anatomy, including variations thereof, should be well known in patients with DS who will undergo a mandibular implant. In addition, anatomic variations in the foramen region and enlargement of the mandibular foramen (MF) may affect the effectiveness of mandibular anesthesia.

Various studies have used panoramic radiography to demonstrate dental anomalies [11], estimate skeletal age [12], or evaluate the periodontal disease and bone loss of DS patients [13]. The aim of the present study is to determine the anatomic features of the mandible such as the mandibular ramus (MR), the mandibular canal (MC), the distance from the MC to the alveolar crest upper limit (C-AUL), and MF of DS patients using panoramic radiography.

## Methods

This cross-sectional study included thirty-four DS patients and thirty four age- and gender-matched control subjects. Eighty-three patients with DS attended the clinics of the Faculty of Dentistry of Akdeniz University for various reasons between January 2014 and August 2016. Thirty-four of the eighty-three (age range: 7–46 years, mean age: 15.5 years; 14 males, 20 females) were included in the study. Participants in this research clearly consented in written to being involved in. Participants who are over 18 years old, gave their own consent. Participants who are under 18 years old or have DS, involved in this study with the consent of their parents. The inclusion criteria was having suitable panoramic radiography for measurements. Exclusion criteria were not having panoramic radiography, presence of motion artefact in panoramic radiography, not having any mandibular molars, having cranial trauma history, having disease that may affect bone metabolism, such as hyperparathyroidism, osteogenesis imperfecta, osteomalacia, diabetes mellitus, osteopetrosis, Cushing’s disease, Paget’s disease, and renal insufficiency.

Atlantoaxial instability is a musculoskeletal disorder estimated to occur in 1 to 2% of patients with DS [14]. Patients who underwent panoramic radiography were separated into two groups (under/over 15 years) to determine the variable effects of the growth and development period on the mandibular morphology. This separation was based on the classification criteria used in the determination of atlantoaxial instability defined by Morton et al. [15] to follow decreasing atlantoaxial instability with increasing age. Although there is no consensus on this age, 15 is preferred for separation so that the groups are balanced numerically.

Each panoramic radiography was taken using a Planmeca Romexis (Helsinki, Finland) at 16 s, 66 kV, and 9 mA. Fifteen patients (nine girls, six boys; age range: 7–11) under the age of 15 years and 19 patients (11 females, 8 males; age range: 15–46) over the age of 15 who underwent panoramic radiography were included in the study. For both groups, a control group was created with an equal number of patients of the same age and gender with the same inclusion/exclusion criteria of DS group who were admitted to the clinic on the same day. If no control group candidate who met the desired criteria was found on the same day, a control group was formed by looking at the previous day and repeating this process until an eligible candidate was found.

### Mandibular measurements

**Fig. 1:**
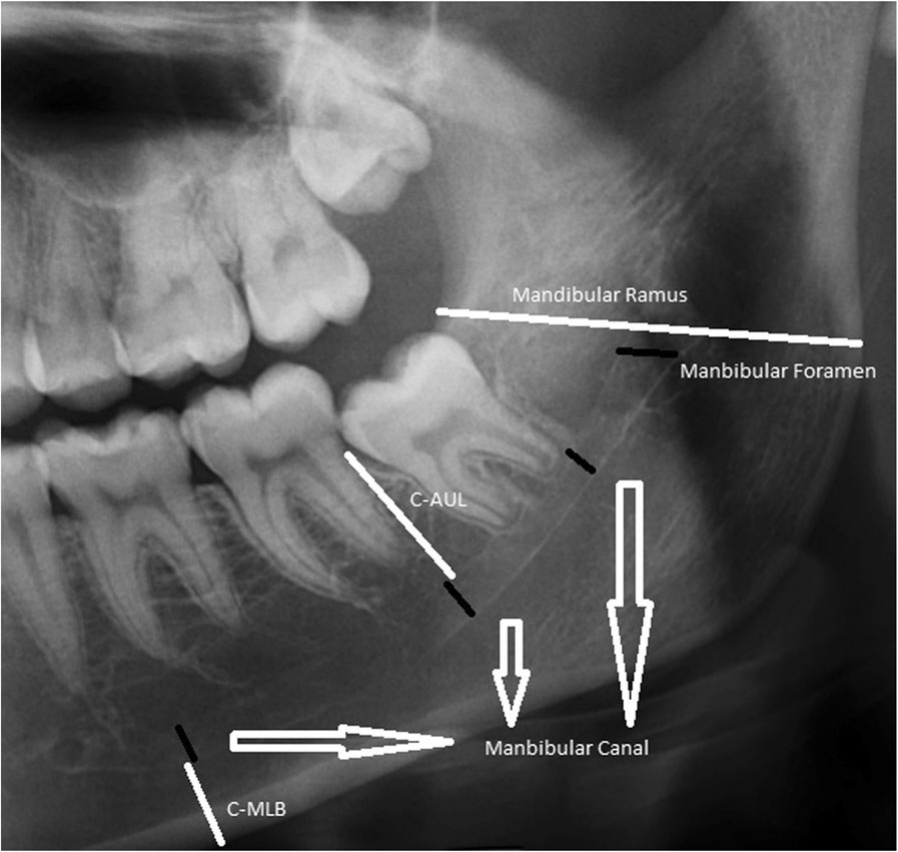
Illustration of measurements made on panoramic radiography

The measurements made were:

1. MF width.
2. Horizontal width of the MR.
3. MC vertical dimension.
4. Distance between the MC and the mandibular lower border (C-MLB).
5. C-AUL of both the right and left side independently in millimeters (mm) unit (According to the Planmeca Romexis guide, the magnification factor in panoramic radiograph is 1,2. Measurements were made based on the known reference objects in the image to avoid magnification in the panoramic radiographs. For this purpose, the calibration tool in Planmeca Romexis software was used.) (Fig. 1).

#### MF width

**Fig. 2:**
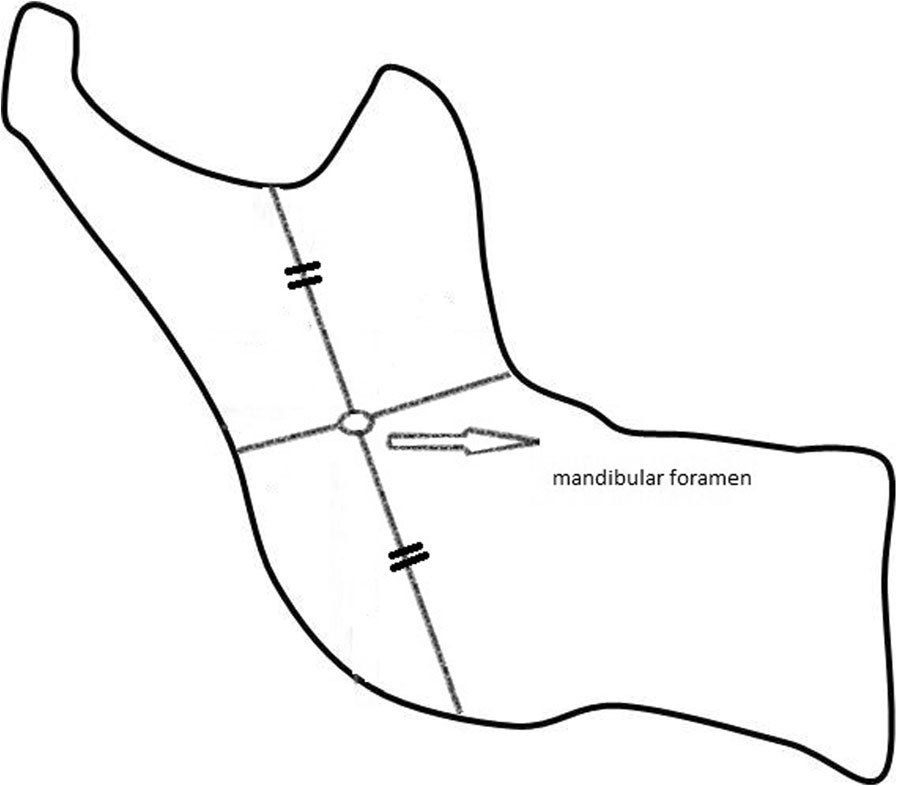
Illustration of the path followed in determining the location of the mandibular foramen

The MF width was measured considering the midpoint of the ramus vertical height between the deeper point of the sigmoid notch and the angulus ramus, taking into account the localization studies performed by Matveeva et al. [16] on dry human mandibles (Fig. 2).

#### Horizontal width of the MR

The horizontal width of the ramus was determined so that the line passing through the foramen was taken as a basis.

#### MC vertical dimension

When the MC, C-MLB, and C-AUL were calculated, the study conducted for localization of the mental foramen and MC by Afkhami et al. [17] was used. Three methods were used for measurements of MC:

1. If there were three molars on the mandible, the MC width was measured at the level of the apex of the mesial root of the first molar tooth and distal roots of the second and third molar teeth. Finally, the average of these three measurements was recorded as the MC width.
2. If there were two molars on the mandible, the MC width was measured at the level of the apex of the mesial root of the more mesial molar and distal root of the more distal molar. Finally, the average of these two measurements was recorded.
3. If there was one molar on the mandible, the MC width was measured at the level of the apex of both the mesial and distal roots of the included molar. Finally, the average of these two measurements was recorded. If there was no molar tooth on the left and/or right side, the patient was not included in the study.

#### C-MLB

C-MLB was measured at the level of the apex of the mesial root of the first molar tooth or the second or third molar tooth, respectively, if the former was absent.

#### C-AUL

C-AUL was measured at the level of the apex of the distal root of the second molar tooth or the first molar or third molar tooth, respectively, if the former was absent.

### Statistical analysis

SPSS 23.0 was used for the analysis. A Shapiro-Wilk normality test, central tendency definition, and skewness and kurtosis coefficients were used to determine whether the data obtained from control and study groups were normally distributed. Box’s M test and Levene’s test were used to test variance homogeneity. The significance of the results was evaluated according to Pillai’s trace criteria when the *p* value of the Box’s M test result was smaller than 0.001. To determine whether the MF, MR, MC, C-AUL, and C-MLB scores differed according to the groups (DS and control), one-way MANCOVA was applied in which gender and age were taken as covariates. The Cohen effect sizes were evaluated as low (0.01), moderate (0.06), and high (0.14) according to the references [18]. A *p* value less than 0.05 was considered statistically significant.

## Results

The results of MANCOVA analysis (Table 1), descriptive statistics of group averages, common effect, gender effect, and effect of the covariate age on the difference between the groups (Table 2) are presented.

**Table 1:** The results of MANCOVA analysis of medium impact according to the general effect, gender and age groups according to the group

Effect	F	SD	p	Partial η 2	Observed Power
Group	23.100	5.128	**< 0.001**	0.474	1
Sex	3.603	5.128	**0.004**	0.123	0.915
Age.Group	19.289	5.128	**< 0.001**	0.43	1

The bold characters expresses significant differences

**Table 2:** Descriptive statistics of group averages and the effect of common effect, gender and age common variables of the difference between the measurements of groups

		Group						
	*DS*		*Control*					
	Mean	SD	Mean	SD	F	Sig.	Partial η ^2^	Observed Power
MF	**4.46**	0.88	4.09	0.61	9.207	**0.003**	0.065	0.854
MR	22.88	2.79	**25.5**	2.42	40.518	**< 0,001**	0.235	1
MC	2.35	0.26	**2.59**	0.34	23.747	**< 0,001**	0.152	0.998
C-MLB	5.04	1.16	4.87	1.27	1.039	0.31	0.008	0.173
C-AUL	9.3	2	**11.92**	2.11	58.571	**< 0,001**	0.307	1
		Sex (covariate)						
	*Female*		*Male*					
	Mean	SD	Mean	SD	F	Sig.	Partial η ^2^	Observed Power
MF	4.19	0.74	4.39	0.82	2.313	0.131	0.017	0.327
MR	23.69	2.89	**24.91**	2.82	8.006	**0.005**	0.057	0.802
MC	2.41	0.26	**2.56**	0.38	8.272	**0.005**	0.059	0.815
C-MLB	4.74	0.95	**5.26**	1.48	8.635	**0.004**	0.061	0.831
C-AUL	10.5	2.08	10.77	2.88	0.506	0.478	0.004	0.109
		Age.Group (covariate)						
	*< 15*		*≥15*					
	Mean	SD	Mean	SD	F	Sig.	Partial η ^2^	Observed Power
MF	4.05	0.59	**4.46**	0.86	10.485	**0.002**	0.074	0.895
MR	23.16	2.72	**25**	2.83	19.105	**< 0,001**	0.126	0.991
MC	2.36	0.24	**2.56**	0.36	16.763	**< 0,001**	0.113	0.982
C-MLB	4.16	0.76	**5.58**	1.14	71.778	**< 0,001**	0.352	1
C-AUL	10.04	1.7	**11.06**	2.82	8.626	**0.004**	0.061	0.83

The bold characters expresses significant differences

According to Pillai’s trace criteria, the group variable (DS-control) was significant in a way that reflects a large effect size (F5,128 = 23,100, *p* < 0.001; Partial η2 = 0.474). The effect of gender as a fixed variable on measurements was significant and moderately effective (F5,128 = 3603; *p* = 0.004; Partial η2 = 0.123). Covariate age was significant in a way that reflects a large effect size (F5,128 = 19,289; *p* < 0.001; Partial η2 = 0.43).

When the main effect according to the group was examined separately according to each measurement, the MF in the DS group was high with a moderate effect (*F* = 9207; *p* = 0,003). MR (F = 40,518; *p* < 0.001), MC (*F* = 23,747; *p* < 0.001), and C-AUL (*F* = 58,571; *p* < 0.001) in the DS group were lower with a larger effect. C-MLB did not significantly differ between the groups, and the effect size was quite low (*p* > 0.05).

When the effect of gender was examined separately according to each measurement, MR (*F* = 8006; *p* = 0.005) and MC (*F* = 8272; *p* = 0.005) were found to be high in males, with a moderate effect. C-MLB (*F* = 8635; *p* = 0.004) was higher in males, with a large effect. MF and C-AUL did not differ between the gender groups, and the effect size was low (*p* > 0.05).

When the covariate effect of age was examined separately according to each measurement, all measurements of the patients in the age group of over 15 were found to be significantly higher than in the age group of under 15, at least with a moderate effect (Partial η2 > 0.06; *p* < 0.01).

## Discussion

Although MF was higher in DS individuals than in non-DS individuals, MC, MR, and C-AUL were found to be low. This situation can be explained by the insufficient growth and development and the different morphological features of the MC in DS patients compared to non-DS patients. Although the C-AUL score was found to be lower in individuals with DS than in non-DS individuals, no significant difference was found in terms of gender. This may be explained by the fact that periodontal diseases are more common in DS individuals than in non-DS individuals and cause alveolar bone loss regardless of gender. No significant difference was found between DS and non-DS individuals in terms of C-MLB score. All measurements included in the study were significantly higher in the ≥15 group than in the < 15 group. In addition, C-MLB, MR, and MC scores were higher in males. These results can be explained by the natural effect of growth and development and gender on anatomical features.

Shalini et al. [19] measured the width of MF at 4.19 and 4.37 mm on the right and the left side, respectively, on dry human mandibles. Ikeda et al. [20] reported that the width of the MC was 4.1 mm in the area near the MF in their study, which was performed using multiplanar magnetic resonance (MRI). In this study, MF width was calculated as 4.1 mm on the right and 4.2 mm on the left side. Although the findings of this study are consistent with past reports, the possibility of differences in the imaging/measurement techniques used and the selected guide points in the foramen measurement may have caused variations in the data.

Despite the reduction in the growth rate in some periods observed in DS individuals in a Turkish study, the study demonstrated that the growth curve was similar to that of non-DS individuals. It has also been reported that the head circumference of DS individuals is lower than that of non-DS individuals, and the head circumference of DS girls is lower than that of DS boys. The low MR and MC scores in DS individuals compared to non-DS individuals and DS females compared to DS males are consistent with the literature [3].

The MC enlarges as it approaches the MF, as shown in the study by Chrcanovic et al. [21]. In this study, the MF was always higher than the MC. The MC was found to be low in individuals with DS, although the MF was higher than that in the control group. This suggests that DS individuals may differ from non-DS individuals in terms of mandibular anatomic features. The fact that the MF and MC were not measured in the horizontal when measured in the vertical may have prevented finding the correct diameter.

DS individuals are typically considered to be skeletal class III [4]. Although C-MLB score was significantly higher in males and the ≥15 group, no significant difference was found between DS and non-DS individuals. This can be explained by the effect of growth and development and gender on anatomical features in accordance with the C-MLB score. However, there was no evidence in this study to support the idea that mandibular prognathism is more common in DS individuals.

Abeleira et al. [22] tried to define the morphology of hard palate in DS with performing some measurements with cone beam computed tomography (CBCT) in axial, sagittal and coronal plane. They showed that the hard palate is narrower in DS than in control group, but the anteroposterior measurements are similar in both groups. Hard palate is narrower in the DS group compared to the control group can be explained by growth deficiency. From this point; in the present study, the lower MR, MC and C-AUL scores in DS compared controls were consistent with the literature. Also Abeleira et al. [22] found no statistically significant differences between males and females with DS for any measurements. In the present study, C-AUL and MF score are showed no significant difference in terms of gender only.

Rodríguez et al. [23] reassessed the dental asymmetry and dental morphologic features in DS in their study. They did not find greater dental crown asymmetry in DS individuals than in the controls contrast past reports which was reported greater dental asymmetry. In current study, there is no significant differences between left and the right side in all measurements and this results is not specified in the statistical results because it is considered normal. Also, almost all dental morphometric measurement scores such as root length and mesio-distal diameter of teeth were significantly lower in the individuals with DS compared controls in the study of Rodríguez et al. [23] These results consistent with the current study in terms of lower MR, MC and C-AUL scores in DS than controls.

Sakellari et al. [6] showed that periodontal inflammation was more common in DS individuals than in cerebral palsy patients and in healthy subjects under 30 years of age [5]. In addition, Amano et al. [24] found that the level of Porphyromonas gingivalis in people with DS over 5 years of age was higher than in non-DS individuals. Corcuera-Flores et al. [13] used panoramic radiographs in their study and showed that DS individuals have a higher risk of marginal bone loss around the implants. The C-AUL score was lower in DS individuals than in non-DS individuals, regardless of gender. This result is consistent with the literature with regard to demonstrating marginal bone loss. Because panoramic radiographs were used in this study, only the height of the alveolar bone was measured. There are no data on clinical periodontal measurements or periodontal soft tissues. Therefore, based on the findings of this study, the low C-AUL score in DS individuals compared to non-DS individuals cannot be explained by only marginal bone loss related to periodontal disease. Growth and development retardation in DS individuals or changes/errors in reference measurement points may also be among the reasons for low bone height.

**Fig. 3:**
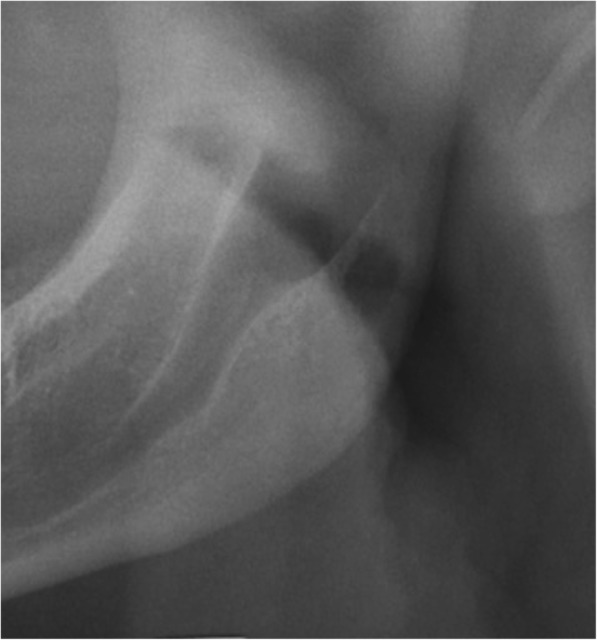
Enlargement of mandibular canal and mandibular foramen on the left side in 29 years old male patient with DS

It is mentioned in the literature that there may be an enlarged MC and MF in patients with neurofibromatosis [25]. In this study, the canal width of patients with DS was significantly lower than that in the control group, while the foramen width was found to be greater (Fig. 3). The fact that the MF of each patient had to be measured at the midpoint of the vertical length of the ramus led to the failure to determine the correct MF width.

The average age of individuals with DS has increased over the years. According to research conducted in different societies, the average age of 9 in 1929 increased to 12 in 1949 and to 50–60 in the 2000s [2]. In addition, some studies have reported the social status of DS individuals [26]. Also, although implant applications in patients with DS are usually performed under general anesthesia [8], local anesthesia procedures have also been reported [9, 10]. When the increase in average life span and social adaptation are considered, it is indicative that there will be a patient profile of DS individuals who will attend clinics in the coming years, and this has increased even more than the expectation of complicated treatment has in the past. Implant-supported prostheses may also be included in complicated treatments. Thus, more detailed information about alveolar bone morphology may be needed in DS patients.

### Limitations

Due to the two-dimensional imaging technique, which caused erroneous results in the study measurements, and the small number of patients included, the study should be repeated in order to confirm the results. It would be useful to create a larger study group and to use a three-dimensional imaging method such as CBCT and MRI, which produce more reliable measurements.

Only the mandible was included in the study because the superposition of anatomical landmarks such as the palatine bone, zygomatic arch, and maxillary sinus in the maxillary region prevented the correct measurement in panoramic radiographs.

## Conclusions

Mandibular canal and mandibular foramen may exhibit anatomical variations in DS. Mandibular morphology especially the alveolar bone level may differ from non-DS due to growth development retardation and/or periodontal diseases.

## Abbreviations

C-AUL: The distance between MC and alveolar crest upper limit
CBCT: Cone-beam computed tomography
C-MLB: The distance between MC and mandibular lower border
DS: Down’s syndrome
kV: kilovolt
mA: milliampere
MANCOVA: Multivariate analysis of covariance
MC: Mandibular canal
MF: Mandibular foramen
mm: millimeters
MR: Mandibular ramus
MRI: Multiplanar magnetic resonance
